# The unheard path: carer loneliness and epistemic injustice in critical and historical view

**DOI:** 10.1057/s41599-026-07113-2

**Published:** 2026-04-09

**Authors:** Fred Cooper

**Affiliations:** University of Bristol, Bristol, UK

**Keywords:** Health humanities, History, Medical humanities, Philosophy, Social policy, Sociology

## Abstract

This article analyses a corpus of longitudinal interview evidence on the experiences of family carers in the UK during the COVID-19 pandemic, and situates them in a longer historical, philosophical, and political discussion. Drawing on feminist epistemology, medical humanities, loneliness studies, and interdisciplinary scholarship on care, it rethinks carer loneliness and epistemic constraint as the mingled products of a series of overlapping abandonments, across short, medium, and long-term temporalities. Discussing loneliness, touch, solitude, shame, and social and cultural invisibility in the stories told by carers about their lives before and during COVID, it then pulls focus to a historical analysis of post-war discourses on care, which simultaneously noted and naturalised the exclusion of carers from emergent imaginings of health and thriving.

## Introduction

The relationship between loneliness and care is well-travelled ground. Family carers – usually defined as people who provide unpaid care for partners, children, relatives, or friends, mostly but not always in their own homes – face a number of significant barriers to healthy relationships, belonging, and community embeddedness. Writing about carer loneliness is a meaningful way of drawing attention to the specific challenges and demands of care, and the ongoing and urgent need for services to provide a counterweight (Long et al. 2017; Victor et al. 2020; Bonin-Guillaume et al. 2022). Loneliness has also been implicated as a contributing factor in carer suicidality (O’Dwyer et al. 2021). Even across a body of valid and useful scholarly work, however, the language of loneliness has been more of resignation than complaint, noting that carers are more likely than non-carers to feel lonely, and positing largely superficial solutions to mitigate those feelings. As Simple Rajrah put it in a 2021 essay, ‘loneliness has come to occupy a more subtly poignant form than an abrasively shocking one—a betrayal smoothened over time and transformed into a living condition’ (Rajrah 2021).

Far from unique to research or activism on care, this reflects a broader academic, political, and third sector investment in loneliness as a form of hesitant or woolly critique, with its ability to provide a real indictment of political and social arrangements largely stripped or syphoned away (Magnet and Orr 2022; Wilkinson 2022; Jones 2022; Cotton 2023; Joffre-Eichhorn and Anderson 2023; Sagan 2023; Cooper 2024). Far more the focus of the present article, it also reflects an interconnected lack of attention to what the deep-seated structural and historical causes of carer loneliness are. While some scholarship and activism increasingly centres systemic and collective problems and solutions, mainstream narratives and resources frequently privatise carer issues, emphasising individual responsibility for managing, coping, or accessing support, or artificially disentangle carers from other, overlapping claims to justice (Lloyd 2006, p. 953; Funk et al. 2024, p. 5). This is particularly troublesome considering how feminised and racialised caring work is, how many carers are themselves disabled, and the impossibility of extricating care from the history and politics of ableism and disability more broadly (Eales and Peers 2020). This problem of clear seeing then has the practical effect of stymying speculative thinking about how carer loneliness might be prevented and reversed, rather than simply managed or ameliorated.

What is needed—and what this article goes some way towards—is a fuller understanding of how carers have been systemically excluded from collective imaginings and aspirations of social and relational health, in concentric circles of neglect, marginalisation, and forgetting; and what the experiential and epistemic terms of this exclusion have been. As one of the research participants discussed in this article put it, carers can often find themselves on ‘a very unseen, unheard path’ (Participant 9, week 12; hereafter P9W12). Attending closely to carer narratives from a critical and historical perspective allows for a timely reckoning with liberal accounts of loneliness and care. In particular, the multidimensional nature of loneliness and its determination by both material conditions and problems of recognition and devaluation enables carers’ experiences to inform a complex analysis which shifts the dial on understandings of how these factors interrelate.

This article begins with an exploration of carer loneliness in the UK, during and before the COVID-19 pandemic. While detailing the particular challenges of providing care in the shadow of the virus and under the conditions of partial quarantine colloquially termed ‘lockdown’, carers used these conversations to narrate longer histories of loneliness, often situating the pandemic in terms of altered continuity rather than absolute change. As much scholarship on loneliness and COVID-19 has revolved around transitions to remote or digital forms of connection, this section will linger—but not, I hope, either normatively or nostalgically—on experiences of interrupted or estranging touch. One significant and under-discussed element of carer loneliness is that possibilities for healthy sociality and healthy solitude are often simultaneously constrained; this problem came into particular contention as the meagre services aimed at providing relief, long identified by carers as inflaming feelings of failure and shame, became even more difficult (and in some cases impossible) to access (Bennett et al. 2020). These considerations, I argue, have sustained—and been sustained by—exchanges, processes, and encounters usefully described in literatures on epistemic injustice and harm. This article contributes, therefore, an interdisciplinary perspective on carer loneliness that is structural and systemic, and which adapts theoretical and methodological practices taken from existing scholarship on care, feminist epistemology, history, and the critical medical humanities.

### Context

Through these discussions, I trace a series of short- and long-term abandonments, in which an extractive approach to carers’ labour was rooted in their simultaneous devaluation as social and civic actors, a phenomenon closely intertwined with capitalist and ableist exclusions of the people they care for. These are as follows: a lack of attention, guidance, and visibility in the immediate context of COVID-19; a wilful dismantling of caring infrastructures and the welfare state over a number of decades; and, drawing on historical evidence, a deliberate exclusion of carers from changing systems of knowledge on the determinants of happiness and health in the mid-late Twentieth century. While some of these processes will be recognisable elsewhere, this article explores loneliness and care in one specific context, the UK. Carers have access to carer-specific forms of material support, such as carers’ allowance, as well as a mixed and uneven slate of state and third sector programmes for respite and assistance. They are also frequently responsible for navigating entitlements for the people they care for, which have been subject to persistent and callous restructuring and contraction in the face of considerable confusion, fear, material disadvantage, and distress (Sense 2025). At the onset of COVID-19, the UK government initially toyed with a policy of mass expendability towards the ageing, ill, and disabled, before implementing public health measures which entailed considerable restrictions to social life with little or no supportive structures in place to allow for significant and prolonged isolation with minimal harm (Philo and Berry 2023). While these measures were inattentive to the relational needs of the public broadly told, they were specifically and pointedly inattentive to anyone whose experience diverged from a homogenised imagining of who that public was composed of. For carers, services closed without notice and sometimes without contact; our interviews were taken shortly after a parliamentary bill was announced, which included the suspension of requirements for local authorities to meet their obligations under the 2014 Care Act (Department of Health and Social Care 2020).

## Methods

This article works primarily from a corpus of qualitative data created through longitudinal interviews with a cohort of family carers in 2020, the first year of the COVID-19 pandemic, as part of an interdisciplinary research and impact project, Caring through Coronavirus, led by Siobhan O'Dwyer. Participants were recruited via existing Patient and Public Involvement groups and the Carer Research Knowledge and Exchange Network (CAREN) mailing list. Interviews were conducted via telephone or digital video platform, and were guided by a semi-structured protocol, developed in consultation with Julia Melluish, a family carer, and an academic team with a significant proportion of researchers with lived experience of giving care. The protocol included standard questions constant across each week of the study, and weekly questions pertinent to recent events and policies; these were devised and structured specifically to make space for carers to articulate their thoughts and experiences, and to foreground what felt particularly important or useful for them. The sample included four male and 11 female carers, ranging in age from 27 to 89 years old. They were caring for children, partners, and parents with a range of conditions, including autism, Down’s syndrome, dementia, chronic fatigue syndrome, Chronic Obstructive Pulmonary Disorder (COPD), and Ehlers–Danlos Syndrome. The majority were providing care in their homes, but one was supporting a parent in a care home, and one was providing care in each of her two care recipients’ homes. All carers identified as heterosexual and white.

Working from verbatim transcripts of the interviews, I coded them for a series of subjects—loneliness, shame, solitude, abandonment, and epistemic harm—which reflected my existing interests as a researcher. This entailed taxonomising an extensive ‘lay language’ of the experiences under consideration, recognising that complex and difficult phenomena are expressed in diverse ways which only sometimes mirror academic terminologies. For example, loneliness and isolation could be (and were) often mentioned outright, but could also be gestured to in more colloquial statements such as ‘no-one gives a shit’ (P2W7). This approach has been more fully theorised in relation to shame, which people name directly relatively rarely; Suzanne Retzinger, for example, has devised a long list of conversational proxies to assist researchers in identifying shame (and anger) without over-reaching or over-writing subjects’ experiences (Retzinger 1995). In terms of epistemic harms, proxies were the sole means of identification; alongside words and phrases such as ‘listen’, ‘heard’, ‘know best’, and ‘ignored’, I paid particular attention to the sections of the transcripts where participants described their interactions with different services, or spoke about broader political and cultural valuations of their work.

These were then analysed iteratively in dialogue with my theoretical reading, a rigorous humanities methodology which has been described at length by Greenhalgh, among others (Greenhalgh 2016). I am an interdisciplinary scholar working in a number of different analytical traditions, each of which surfaces or recedes in response to the texts I engage with. Broadly speaking, I apply a form of critical discourse analysis which approaches testimonial data as a form of narrative produced within the particular circumstances of the interview, but takes pains not to over-interpret the hard-won insider knowledge that participants articulate. My combination of interview data and historical sources follows my longstanding commitment to demonstrating the past dimensions of present experience, and the historical component of the research was undertaken in response to the themes identified in the interviews and the literature.

Designed to inform policy and practice responses to the pandemic in real time, Caring through Coronavirus was an attempt to insert experiential knowledge into a rapidly changing epistemic economy, as viral risk and strict limitations on mobility, tactility, and sociality significantly altered the experience of giving and receiving care. At the time of writing in 2024, as COVID-19 has lost ground as a political and public health priority, the value of the evidence that Caring through Coronavirus created—written transcripts of weekly, semi-structured interviews with 15 carers across 12 weeks—has shifted significantly in tenor. From a historical perspective, it is of vital importance that analyses exist of how the pandemic was felt and known. As a number of scholars have pointed out, COVID-19 acted as a ‘breaching experiment’, creating affordances for older tensions and fissures to move into greater view (see e.g. Scambler 2020). In this instance, the pandemic—and the UK government’s inept and neglectful responses to it—created an additional layer in the complex reality of carer loneliness and abandonment, offering a useful opportunity for scholars of care to tack between acute and chronic temporalities (Grycuk 2022). In work which might seem, at first glance, to be about care in the context of almost complete physical quarantine, we can also spend time with the personal and political histories that precede, weave through, and outlive those specific moments of crisis.

Indeed, in the final section of the article, my focus shifts to a relatively brief historical interrogation of narratives on care in the second half of the Twentieth century. Although this represents a distinct change in evidence and tone, it should be understood as a continuity in subject and argument; the historical developments discussed are of vital importance to the themes of the piece as a whole. The placement of this section—and a wider layering of context throughout—are deliberate choices on the part of the author, pushing against disciplinary hierarchies which relegate historical research to the background, the part that sets up the implicitly more important analysis to follow. In this article, the historical work is the closing point, the argument that changes how the reader thinks about the nature and intention of the harms described.

### Loneliness, reciprocity, and touch

Loneliness plays a complicated—and sometimes conflicting—role in carers’ inner lives. Alongside (and often above) anxieties over how the caring role can stifle important relational opportunities, carers worry about the people they care for; the work of care is framed and shadowed by loneliness, whether as an active feeling which carers try to dispel, or a possible or imagined consequence of reducing or withdrawing their care. A 2021 study on dementia, carers, and pandemic loneliness, for example, noted that ‘carers of those with dementia may prioritise providing care that protects the person with dementia from loneliness at the cost of experiencing loneliness themselves’ (Perach et al. 2022, p. 521).

If care in its broader set of meanings is a vital antidote to the care-less systems that make people feel isolated and alone, then family carers provide this function—among many others—for millions of disabled, ageing, and chronically physically or mentally unwell people (Feng and Astell-Burt 2022). Far from inevitable, their isolation and exclusion can be understood in the first instance as a facet of extractive capitalist ableism, which demarcates between people who can and can’t be profitably exploited as workers; i.e. without making costly adjustments to facilitate their presence in the labour force (Russell 2001, p. 90, p. 92). In the second instance, loneliness is produced and amplified by the prolonged liminality of deinstitutionalisation, in which ‘widespread closures of the institutions that warehoused disabled people [took place] without an allocation of adequate resources and services to enable them to live independently’ (Russell and Malhotra 2002, p. 216). In the UK, a ‘revised philosophy’ of community care consequently emphasised the unpaid caring work of family and friends, and reimagined social contacts as providers of care, preferably within private homes (Heaton 1999, p. 763). As historians such as Pat Thane, Katherine Holden, and Chris Harris have examined, the creation of the welfare state did not significantly alter women’s responsibilities for providing complex care (Thane 2000; Holden 2001, p. 137; Harris 1994, p. 54). Instead, a mixed economy of care and welfare relied extensively on their labour, preventing many in the process from living full social, emotional, economic, and relational lives (Glendinning 1992; Nicoriciu, Elliot 2023). Where ageing and disabled people could and should have been supported to be embedded and valued civic and social agents, they have been atomised instead into the overloaded, isolated, poorly-braced, and porous dyad of carer and cared-for that emerged through these primarily financial calculations and contradictions (Carson 2024).

For Liz Lloyd, the ‘differentiation of carers’ needs from those of people in need of care and support’ is a ‘self-defeating objective’, not least because so many carers are disabled, chronically ill, and themselves in receipt of care, frequently from the people they care for in turn (Lloyd 2006, p. 946; Eales and Peers 2020). The neoliberal individualisation of responsibility for meeting these needs carries—as loneliness does—extensive and ingrained connotations of personal failure (Wilkinson 2022). In a context where loneliness is privatised and purposively uncoupled from political causative stories, it also keeps carers caring in the most difficult, lonely, and harmful circumstances. While many carers see what they do in relational terms – for example, as an extension of love or the unavoidable result of familial obligation – they also internalise a sense of themselves as accountable for a particular kind of intimate affective work, responsible for preventing, managing, and containing difficult emotions (Lynch 2007).

The carers in our work articulated anxieties around this role in a number of ways. At different stages of the study, concerns over the people they care for feeling lonely or disconnected surfaced in the interviews; difficult conversations with older children about how many friends they have, or, as public health restrictions came into effect, the painful observation that crucial peer support systems weren’t making the necessary shift online (P9W11; P15W1). However, it was in conversations around touch – increasingly, in the spring and summer of 2020, a fraught and vexed thing – that carers made space to reflect on their own relational needs, shifting discussions of loneliness and care into a more mutual register (De Luca 2020). While our interviews took place in one of the more acute phases of quarantine, other research suggests that carer experiences of loneliness became even more pronounced as the pandemic progressed (Webb et al. 2023, p. 10).

At times, speaking about touch followed more well-travelled narratives of concern, as government admonitions to keep distance landed in jarring and awkward ways. Often, embodied experiences of connection had a heightened resonance, and their withdrawal was felt more keenly—and grieved—as absence or loss. For example, one parent spoke at length about how their son often required physical cues to engage; physical distancing meant that he was unable to be tactile in the usual way, which excluded him further from conversation (P12W10). This reflected a broader problem of normative imagination throughout the pandemic, in which a sustained inattention to diverse needs, circumstances and experiences meant that some public health directives worked a peculiar kind of violence, imposing rules which altered the lives of marginalised groups in unbearable ways (Cooper, Dolezal and Rose 2023; Emadi 2024; Blunt et al. 2024).

A second carer spoke about the protracted experience of caring at a distance for their son and their father; neither lived in the same household, so they were expected to maintain a strict physical quarantine. This interjected painfully into reciprocal practices of touch, practices which sustained both carer and cared-for. Worrying constantly about loneliness and suicidal thoughts, their concern manifested in a series of bodily complaints; as they put it, ‘physically and mentally, it’s all going on underneath’ (P13W3). Across a number of conversations, touch emerged as an area of serious anxiety; for example, their father reminded them of ‘all those animals without being given a real mother who withers away even though they’re being fed and watered, they need the touch’ (P13W2)^1^. Tactile by disposition, their son also found physical distance difficult to cope with. In the sixth week of the study, they improvised a makeshift compromise:

He’s got his old cuddly toy out of the cupboard. It’s an owl. I’ve got a cuddly teddy that somebody gave me after my husband died. So we sit and talk and cuddle our respective toys, which is quite nice too because it means that emotionally you can feel there’s something to cuddle, because the whole business of not having anybody to touch under any circumstances is really hard, for him and for me (P13W6).

The use of proxies to counteract unsettling experiences of embodied estrangement is striking, particularly as—in the case of the carer—they rehearsed older feelings of grief and loss, feelings which, much like loneliness, have an under-acknowledged material history and culture (Bound Alberti 2018). Towards the end of the interview process, when supportive ‘bubbles’ (small fixed groups in which proximity and touch were permitted) had been introduced, they revisited their earlier plight with a new cheerfulness: ‘the power of touch is incredible, not having had any touch for all those weeks, and just being able to touch somebody is wonderful. He needed it, but so did I’ (P13W11).

In a similar story of suffering and restitution, one of our opening interviews met the carer in question partway through a period of quarantine, as she isolated from her clinically vulnerable husband despite continuing to provide physical care. This resulted in a kind of acute alienation, as the care she gave felt instrumental, routine, and depersonalised:

The type of care has changed because I’ve, how can I put it, I’ve become more of a carer than a wife. I put a mask on if I go near him. I touch him with plastic gloves on. I help him shower as well. We’ve got a walk-in shower, a wet room, so I have to help him shower. So that’s the only sort of contact we’re having with each other. And I miss it.

The interviewer then asked her: ‘How does that feel for you in your person, and in your body, I guess, not being able to touch each other?’ She responded, ‘It’s horrible. This is where I get teary, sorry’ (P4W1). A week later, when the quarantine was over, her mood was significantly better; asked about the change in her situation, she said: ‘It’s what makes you human, isn’t it, being able to just hold on to each other and show your love for each other’ (P4W2). Touch without space for feeling – care through plastic, becoming clinical—dehumanised them both (Eales and Peers 2020, p. 4).

In each of these accounts, an emphasis on touch allowed carers to foreground their own needs and emotions, in a necessarily complex imagining of care as simultaneously process and source of comfort and consolation; underscoring, along the way, the wider insight that loneliness can be just as much about restricted opportunities to *give* care, support, and meaning (Brownlee 2016). Of course, this was also a profoundly ambivalent thing. Carers narrated their own loneliness in diverse ways, building causative stories which moved between different kinds of personal and public history, and between long and short temporal scopes. In an understandable preoccupation with the immediate context of the early pandemic, some comments can be read as straightforward responses to public health measures which relied, very literally, on isolation; one carer noted that ‘the overall effect of no social contact is beginning to affect everyone’ (P6W10), while another spoke about the ‘suddenness of how everything had to change and the feeling of total isolation’ in the first weeks of restrictions (P8W12). For others, their relationship with loneliness was deeper, pre-existing even their caring role; as was the case with a carer whose ‘sense of not belonging, being different, and isolated’ collided with caring—and, later, caring during a pandemic—in complicated ways (P11W9). Other comments alluded to loneliness more ambiguously; one carer, for example, excused their confusion over interview dates by explaining that ‘apart from speaking to you, I don’t think I’ve spoken to anybody all week’ (P2W4). This crystallised an unintended (and not unclouded) function that the researchers on the project sometimes began to take, that of the listener and confidant, ourselves providing an ad hoc form of care (Easter et al. 2006).

Frequently, though, the carers interviewed worked from a politicised and contextual understanding of loneliness as a problem fundamentally related to their role. Discussing the government’s response to COVID-19, one explained that you ‘almost get the feeling you’re being imprisoned at home and you’re more isolated *than ever*’ (P8W7). This formulation hinted at a longer story, and they returned to the theme two weeks later: ‘as carers you sometimes can feel so isolated and that others don’t care for you’ (P8W9). Another spoke candidly, and with humour, about their realisation that they have no family and friends: ‘No-one gives a shit. I’ve spoken to a couple of people I hardly know, but it’s different when you’re my age, and you’re a carer, you’re stuck at home a lot… If I were by myself, I’d probably draw a face on a baseball and start talking to it’ (P2W7)^2^. Scambler’s description of COVID-19 as a ‘breaching experiment’ offers one way of thinking about a secondary form of narrative breach that occurred within and around the pandemic (Scambler 2020). Stories that began from a description of emotion and experience in a particular time and space quickly overspilled their bounds, winding backwards at times over decades. Loneliness could arrive acutely and abruptly, in the headlong rush from one life to another; in the aftermath of a partner or parent’s debilitating and sudden accident or stroke, for example. More usually, it was incremental and slow, accompanying a gradual narrowing of social worlds (Silverman et al. 2020, p. 337). Temporalities of care can be muddy and strange, with identification as a carer—a prerequisite both for accessing support and for crucial shifts in self-knowledge—commonly solidifying months or years after the work of care has significantly begun (Urwin et al. 2022), if at all (Larkin and Milne 2014, p. 26). Indeed, identifying as a carer and beginning to access resources have frequently been framed as antidotes to isolation (Funk et al. 2024, p. 5). One parent carer described the process of watching their child grow apart from others, and simultaneously losing their own friendships with other parents:

There were things that started to happen, like the people that we were friends with who had children the same age, were gradually beginning to go down different paths because their children were different. And it’s only to be expected, really. So I think when we started feeling very isolated, and I think you as a carer then have to start coping with that kind of isolation… you do tend to lose touch with the world. It’s a bit like an onion: you have these layers peeled off you until in the end you’ve got quite a raw bit in the middle (P8W1).

There are a number of things to take note of here. Their emphasis on coping kept the onus directly on them; loneliness is present as an ‘expected’ challenge of providing care, and the carer’s responsibility to successfully manage and mitigate. This can be interpreted in two ways. On the one hand, it reproduces a neoliberal logic of private problems and private solutions, with a far wider purchase in how the causes and solutions of loneliness are discussed (Wilkinson 2022). On the other hand, it perhaps also represents a realistic response to a context where, through the application of these neoliberal and ‘austerity’ logics to the welfare state, what mechanisms for support there were had been deliberately and ruthlessly hollowed out (Russell and Malhotra 2002, p. 216; Stenning and Hall 2018).

In their reference to having ‘layers peeled off you’, this carer also situated loneliness as a process of attrition, with some integral part of them as attenuated and inscribed; a process, in ‘peeled’, not without intimations of external culpability. By using the word ‘we’ to refer to the isolation of both carer and cared-for, this testimony further troubles the assumption that the default unit of loneliness is necessarily the single person. Important insights on isolated families and processes of ‘social encystment’ affecting entire households in the mid-twentieth century have enjoyed comparatively limited traction since, reflecting a liberal emphasis on individuals which obscures the myriad ways that people become—and are—lonely together (Pearse and Crocker 1943, p. 248; see also Tietjen and Furtak 2021).

### Solitude and shame

Understanding carer loneliness, here, requires a commitment to thinking critically about how problems of being and feeling alone manifest within and across the caring relationship, and how this is bounded by structural ableism and the political economy of care under ‘austerity’. Carers’ frustrations around access to solitude can offer a useful insight here. While interventions on loneliness usually revolve around disrupting isolation, psychoanalytical work has long emphasised that loneliness can also stem from an unhealthy or distorted relationship with solitariness, an experience which might otherwise be generative or pleasant (Winnicott 1958; Storr 1988). People who feel lonely can be excluded from the goods of solitude, but the relationship with care is more complex; more often, carers are excluded from these goods by the practical difficulties of securing time alone. Always a consideration, this entered into a heightened state of contention in 2020 as existing (and limited) avenues for respite swiftly retracted (Bennett et al. 2020). Experiences in the UK closely mirrored what Ginsburg and Rapp observed in New York City around the same time: a melting away of the programs, services, and support staff that sustained and structured a number of different goods, carer solitude among them (Ginsburg and Rapp 2025, p. 167). This was accompanied by a tightening of the informal bounds of care, contracting and pressurising sociality and solitariness even further by inhibiting ad hoc help from family and friends. Ginsburg and Rapp describe an ‘overwhelming sense of vulnerability and loneliness as the usual community supports crumbled’ (Ginsburg and Rapp 2025, p. 180).

Although they are rightly conceptually distinct, the spaces between loneliness and solitude are ambiguous, porous and fluid, and the two can often co-exist in the same moment (Davies 1996). For one carer, the pain of loneliness and the need for solitude shared more or less a single breath: ‘I’ve just felt really isolated this week… I just can’t escape, can’t get a minute to myself, and I feel really bad for feeling like it’ (P12W3). Alongside speaking to a phenomenon which is distinctly under-explored—healthy solitude, in and of itself, as a direct antidote to feelings of isolation—this testimony drew together two important strands, each of which bears spending time with. That carers frequently need time alone—or, at least, away from their caring responsibilities—is widely acknowledged, and provides the rationale for a number of services aimed at offering respite (Greenwood, Habibi, and Mackenzie 2012). Alongside building a framework for carers to socialise away from the person or people they care for, however, these services can also be interpreted as providing a (hesitant and faltering) infrastructure for solitude. Accessing respite, then, should be understood as closely implicated in how carers negotiate the harms and comforts of solitariness. Solitude also has a distinct politics, and—particularly here, but also in other experiences—an observable political economy (Vincent 2020). The calculated exclusions that isolate disabled people and the people who care for (and are cared for by) them simultaneously spoil their solitude, making aloneness an unhappy and impoverished thing even when it isn’t withheld.

This carer’s reference to feeling ‘really bad’ about needing time alone introduces another dimension, carer shame. Beyond limited discussions of ‘courtesy stigma’, which chart the effects of a kind of secondary ableism, carer shame is another area yet to be adequately theorised, as are the relationships between shame, self-disgust, and different experiences of aloneness (Vasileiou et al. 2017, p. 8; Ypsilanti 2018; Jeong et al. 2020). Work on ‘shame-proneness’ emphasises that particular populations, usually those subjected to chronic shaming through, for example, white supremacy, ableism, or queerphobia, often inhabit fundamentally altered relationships with shame (Dolezal 2022). There is a good argument for applying many of these insights to carers. To begin with, caring is shamed work (Lynch 2007). While family carers are disproportionately women, men’s experiences of giving care are also coloured with systemic misogyny and contempt for labour culturally devalued as feminine (Chatzidakis et al. 2020, p. 890; Willis et al. 2020). Within this context, caring also carries a number of heightened possibilities for shame and self-recrimination. Some of the carers we worked with spoke at length about the guilt and shame of important decisions over care, or the painful acknowledgement of hostility or resentment; the long and hard labour of caring had forced them to discover things about themselves that—entirely wrongly—made them feel morally compromised, selfish, or ashamed (P2W6; P3W3; P11W9; P12W3; P5W11).

If we are all, as W.E. Douglas Creed put it, ‘swimming in a sea of shame’, then there are further depths and dangers to the labour of care which move between the chronic and the acute, the disremembered and the hyper-visible (Creed et al. 2014). For one carer, deeper feelings of being ‘on the margins, on the fringes’ were crystallised, before the pandemic, by a fraught and exposing shame encounter, an experience which drew much of its traumatising power from a jarring shift between social invisibility and public scrutiny. Taking her son around town, she described an instance of profound humiliation when a stranger shamed them both over a seizure in a public space:

It was a hot day, and he suddenly had one of the unusual seizures that manifested in him behaving very strangely. And people who don’t know look at it as behavioural, and we have had the police called out, for instance, a disturbance in the past. But anyway, on this particular occasion, he came over with one of these seizures… [and somebody] came by and made quite a wide berth, but she made it quite clear… ‘Tut tut’, she was making those very disapproving noises, and it was dreadful (P8W10).

Coming into public view—particularly in a way that was shaming, isolating, and that she had no control over-widened the gulf between her and a community she felt increasingly alienated from. The stranger who shamed the carer and her son policed ableist norms of behavioural and bodily order, which grew from and sustained capitalist logics of exclusion (Russell 2001). These ableist logics guide and frame experiences of giving and receiving care in less perceptible—or more routinised—ways, but they also interject violently and personally in disciplinary episodes that use shame as a means of asserting what kinds of bodies are welcome in public, and under what terms (Ingram 2025). This carer experienced a particular—but by no means uncommon—iteration of ableist shaming; the bystander’s disgust at her son’s seizure was redirected at the person she deemed (able to be) responsible.

This matters particularly here because the grudged emotional and epistemic work of petitioning services for solitude takes place from a shamed—and shame-prone—position, with a number of significant consequences for how carers are able to be alone. Throughout our interviews, solitude took shape as something which was coveted, commodified, and freighted with shame; this echoes earlier findings on carers’ feelings of selfishness around needing time alone (Long et al. 2017). Asking for help carried painful connotations of failure, bound up further with the difficult admission that they needed time away from the people who relied on them. As one carer put it, ‘any option for us to get any form of a break is non-existent, or you’re going to be shamed if you feel you need to take it, or you’ve got to ask for it’ (P10W7). Another noted that ‘you need help, but you’d rather have somebody else offer it than you have to ask for it. Because asking for it means that you’re admitting that you need it, and when you are normally the person who does the helping, and you’re not the one who seeks the help, it’s really hard to ask’ (P13W3). In a long, reflective, and multi-layered testimony, one carer set this problem out in a language indebted to wider literatures on shame:

[Asking for help] just feels so self-indulgent. It really does… one of my huge fears, my toxic shame triggers, is that I wear people down and that they’re going to eventually just start tutting and get fed up with me… I think that shame and guilt cripples human beings. And I know that it has totally crippled me. I think we’re very good at shaming others for things that we don’t feel that they deserve. So in turn, it creates a culture in society where we don’t ask for help… you become secretive with things, or you just feel like I do, I have to go to extreme measures in order to justify some support (P11W11).

Although taking place in the acute abandonment of the early pandemic, in which services retracted rapidly to more or less nothing, these were grievances years in the making. COVID-19 transformed experiences of—and responses to—solitude for many people, but this process was unequal, loaded, and vexed; as Barbara Taylor and David Vincent argue persuasively and at length, the experiential and intellectual history of solitude has always been pressurised and contorted by questions over who is allowed to be solitary, when, where, and how (Taylor 2020; Vincent 2020; Taylor 2024).

### Extractive abandonment and epistemic harm

We can usefully understand shame as a crucial mechanism of a particular kind of systemic abandonment, a fuller theorisation of which is vital for understanding and contextualising the testimonies detailed above. The carer who felt ‘on the margins, on the fringes’ implicated a kind of quasi-systemic estrangement from a community which might otherwise have been a source of support and belonging; as the cultural and social geographer Eleanor Wilkinson reminds us, however, languages of community failure can be just as misleading as languages of individual responsibility, obstructing and drawing the sting from a feminist analysis of loneliness as a structural condition bounded by political systems (Wilkinson 2022). Thinking critically and historically about abandonment shifts focus to how this carer, and many others, have been placed in harm’s way by a long economic and medical reliance on their unpaid, unseen, and shamed labour. Their abandonment was constituted from—and relied on—extraction, with loneliness and shame written in.

Carers narrated this abandonment in a number of different ways, moving between the acute conditions of the pandemic and longer processes of forgetting and effacement. In a notably forceful testimony, one carer—whose sense of abandonment was particularly pronounced—commented that they felt:

More and more isolated from society as a whole because certainly the politicians I feel are treating carers as being very expendable, not understanding their role, not even understanding the terminology of what an unpaid carer is… It does make me feel like that, as if you are not appreciated, but I know because I look at it from the bottom up, otherwise I would get so angry… I would put all my energy into feeling angry and vindictive, and I suppose almost full of hate, I’d put it that strongly (P8W10).

They also set out feelings of what might be termed iatrogenic loneliness, rooted in a lack of contact from their local surgery: ‘I’m still totally disgusted, and I feel more isolated by GPs than I ever have done. GPs in general, never having got in touch, I feel more isolated from them than from anybody in society’ (P8W10; Cook et al. 2021). If not always replicating their intensity of feeling, other carers echoed similar sentiments of abandonment and forgetting, although the identity of the antagonists was sometimes unclear:

I still think we are a forgotten society. If you stopped 100 people in the street and said, ‘Are you aware that people do this on a daily basis and try to fit work in?’ 90 per cent would say, ‘I didn’t know that happened’ (P1W12).

Carers of people who stay at home are always forgotten; they’re the forgotten bloody army (P2W10).

We’re stuck in our home, and nobody knows. But as far as the community is concerned, we don’t exist (P4W12).

There’s very little out there that is looking out for people who are caring. And I think at the moment, with all the social isolation, carers are probably very isolated, and there is no break (P9W12).

You suddenly don’t hear from anybody, we’re now in our fourth week, you kind of feel like you're abandoned, I suppose (P10W2).

We are a forgotten group, really (P12W1).

I think they forgot us. I think they were too busy telling people to stay in that they forgot about people. They forgot about carers, and they forgot about parents who care. They didn’t think one iota about them at all (P14W12).

This process of forgetting had a number of temporal layers. In the immediate term, several of these comments spoke to the more or less complete cessation of support and guidance in the wake of government-mandated quarantine measures, which were unable or unwilling to comprehend that specific groups would require particular attention or be disproportionately harmed by the homogeneity of restrictions (Grycuk 2022). Rather than a new or inexplicable phenomenon, however, this was interpreted as a symptom and sign of a broader lapse in cultural and political memory, a withheld acknowledgement that was far more than just salt in the wound, but a crucial component of carers’ social and civic alienation. There are glimpses of this in work by Konstantina Vasileiou and her research team, as one interviewee admitted that she was experiencing a form of loneliness that was linked to a lack of recognition of her role as a caregiver (Vasileiou et al. 2017, p. 7). Without dismissing or minimising the important gains made by academics, politicians, and activists in making carers more visible over recent decades (Larkin and Milne 2014, pp. 28–29), this underscores that these gains are both incomplete and fragile to fluctuations in political and public imagination.

Carers’ experiences of pandemic abandonment emphasised, sometimes bitterly, the contradictions of their role; disregarded and ‘kept in the dark’, but simultaneously indispensable to the national economy and the continued functioning of the National Health Service (P8W7; P1W1; P15W12). In their 2022 book *Health Communism*, Beatrice Adler-Bolton and Artie Vierkant offer the term ‘extractive abandonment’ to map out how capitalism makes money from a surplus class framed as unprofitable (i.e. as workers) in other ways, including for-profit care homes and lucrative (and arguably ineffectual or harmful) therapeutic industries (Adler-Bolton and Vierkant 2022; Russell 2001, p. 93). This is a distinct argument which I have no intention of co-opting or diluting, but it captures something important about how groups can be simultaneously abandoned and exploited (in this case, in the considerable economic reliance on carers undertaking their labour unpaid). In the UK, the mythos surrounding the British National Health Service (NHS), particularly pronounced during the pandemic, introduced another dimension that shifted rhetorics of carer indispensability and sacrifice into a parallel ideological register; in addition to saving money, they were helping to ‘protect the NHS’ from becoming overwhelmed (Backhouse 2023). Liz Lloyd has written about the counter-productiveness of carers and their organisations adopting and internalising this language of cost; when the economic savings that carers offer are the logic of value, governments are disincentivised from providing support beyond the bare minimum needed to keep them caring (Lloyd 2006, p. 952). Lloyd’s insistence that a politics of carer recognition has been allowed to efface arguments over more sorely-needed material assistance is vital, but the sentiments that carers expressed over invisibility and forgetting nevertheless suggest that such a politics was directly meaningful to them (Lloyd 2006).

Exemplified by ‘clap for carers’, a strange and conflicted phenomenon, widely taken up, of clapping and banging pots and pans in appreciation at 8 pm on Thursday nights, a number of our participants also noted that a public lionisation of ‘carers’ effectively collapsed family carers with healthcare workers, eliding their role in the process. This introduced a new dimension to their invisibility from public discourses on collective contribution at a moment of crisis (P8W10; P15W12; Wood and Skeggs 2020; Cooper, Dolezal and Rose 2023; Higgins and O’Leary 2023). As Andreas Chatzidakis and his collaborators have identified, the ‘discursive explosions of care’ in the first months of the COVID-19 pandemic saw it become ‘the buzzword of the moment, its meanings draining away in its constant evocation’ (Chatzidakis et al. 2020, p. 889).

The problem of how carers might be *known* by others raises significant questions over the epistemic dimensions of the matters under discussion (Brown 2021, p. 56). Several carers framed their invisibility specifically in terms of an inability to speak (and be listened to), or shape wider systems of knowledge; one recalled seeing ‘something on Twitter where somebody said, “stop calling us unseen”… We are ignored! [laughs] I thought that was quite good’ (P5W10)^3^. Observing that ‘support hasn’t been out there the whole time’, another reflected that ‘it’s a very unseen, unheard path that people just get themselves on’ (P9W12). These carers were deploying what we might think of as a lay language of epistemic injustice, a conceptual framework set out in 2007 by the feminist epistemologist Miranda Fricker, and widely developed, nuanced, and adapted since (Fricker 2007; Kidd, Medina, and Pohlhaus Jr. 2019). This can be usefully understood as injustice done to us in our capacity as epistemic agents, as people who form knowledge, both privately and collectively, and attempt to share it effectively and without unjust constraint in a variety of professional, social, political, legal, and medical contexts (Heggen and Berg 2021). As a burgeoning literature on the epistemology of caring has identified, carers occupy an ambiguous and unusual position, both as subject to processes and instances of epistemic injustice themselves, and as interlocutors for people whose epistemic agency can be far more severely constrained (Hodge and Runswick-Cole 2017; Mazanderani et al. 2019; Brown 2021). Russell and Malhotra note that this relationship is not unclouded: experiences and interests don’t always align neatly, and some carer organisations can inadvertently contribute to these epistemic constraints by undermining disabled people’s advocacy on their own behalf (Russell and Malhotra 2002, p. 217).

As one carer put it, people with learning disabilities risked ‘receiving really bad care and dying of things because very often they are not listened to if their parents or carers are not around’ (P5W10). Another elaborated the same point, with the caveat that parents and carers might not be listened to either:

I think the lack of exercise made him very constipated and he suffers a lot from constipation, so we had to up his medication for that… but of course constipation is one of the causes of death of people with a learning disability because it goes untreated and then the bowels tend to rupture… but it does go to show, doesn’t it, that a lot of things that carers talk about that they say really matter, quite often professionals tend to say [laughs] “you’re imagining it.” It’s something that carers always tend to suffer with: “you’re an anxious mother”, or “that doesn’t make any difference”, and “we know best” (P8W5).

This is an almost textbook case of what Fricker termed testimonial injustice, where prejudices about the speaker restrict their agency in communicating relevant and valid knowledge, in a context with significant potential for harm (Fricker 2007). The language of anxious motherhood established a credibility deficit (and reinforced a credibility surplus on the part of the doctor), a crucial component of testimonial injustice, on three interlinking grounds (Carel and Kidd 2014, p. 530). Being ‘anxious’, the carer quoted above was cast as an unreliable interpreter of truth, overexcited, labile, not in control, liable to worry over nothing; being a ‘mother’, she was simultaneously gendered and stripped of professional expertise (having been a nurse before her unpaid caring responsibilities became too great). Similarly, seeking medical care for the post-COVID symptoms of the person they cared for, another carer reflected that ‘even before the virus, doctors were ready to dismiss things’ (P4W9).

Other carers described interactions with medical professionals that were attentive and capacious, practising a form of epistemic care, even within the narrow constraints of brief appointments (P9W4). Nevertheless, descriptions of what Louise Isham and her co-writers describe as ‘contingent and limited credibility’ in clinical encounters still speak to, as one participant put it, ‘huge injustices in the healthcare system for people who are ill and people who are carers’ (Isham et al. 2020, p. 87; P15W10). At times, these identities converged, as was the case for a carer who went to their GP because of the ‘stress and strain of it all’, and found themselves having to defend a contextual explanation of their experience: ‘I said to him [the GP] I don’t at all think I am depressed or of low mood, because I can go out and have a really good time… sometimes I just get home and I think oh god, I can’t even go back in the house because it’s just… the weight of all the jobs and all the stuff that has to happen, particularly for the boys, is exhausting’ (P10W10). Although her analysis has been troubled since, Melinda Hall theorises a relationship between ‘obscured social construction’ and epistemic harm, in which the naturalisation of specific kinds of experience—in this instance, the misapplication of ‘depression or low mood’ to a loaded and contingent feeling of stress and exhaustion—stands in the way of epistemic practices necessary for the realisation of justice (Hall 2017; Degerman 2023). While beyond the scope of the present article, there are elements of this problem in representations of carer loneliness as inevitable, or seemingly innocuous formulations—such as ‘susceptible to loneliness’—which paper over the political mechanisms by which carers are systemically isolated (Willis et al. 2020, p. 311).

Beyond interactions with medical services, carers reported exacting and confusing epistemic exercises in a number of other contexts, usually in the pursuit of support they were entitled to. Set within a wider logic of resource scarcity heightened and deepened by ‘austerity’, the deliberate, wilful, and unnecessary attrition of the welfare state (Stenning and Hall 2018; Chatzidakis et al. 2020, p. 889), mechanisms for financial support, respite services, and other necessary benefits were experienced as fickle, withholding, and hard to understand, and navigating them was a matter of constant struggle in terms of forming, articulating, and communicating knowledge. A number of carers built a picture of assessment procedures which returned unjust results by default, which then collapsed—frequently undefended—after gruelling processes of appeal (P1W1; P9W2). These experiences describe a clear iteration of the opaque institution identified by Havi Carel and Ian James Kidd, in which opportunities for testimonial—and consequently, here, material—justice are stifled by the systematic exclusion of users from knowledge over the inner workings of the services they have to engage with, which are also felt to be ‘consistently and complexly negative’ (Carel and Kidd 2021, p. 475). Carers were caught in a particular epistemic bind, as access to entitlements turned on the—often seemingly deliberately—hard-earned knowledge of what was out there and how it could be successfully obtained: ‘we could have had a Motability car ten years before if only the fuckers had taken a proactive approach to tell people what they’re entitled to, rather than the catch-22 other way round’ (P2W12).

The epistemic dimensions of carer experiences of service use matter profoundly, not least because these were the interactions that often made them feel particularly marginalised or alone. Epistemic injustice has been theorised, not always cleanly, as the epistemic aspect of social and political injustice (Isham et al. 2020, p. 82; Byskov 2021). Carers are not, of course, a homogeneous group, and are subject to a raft of intersecting injustices and abandonments along lines of race, class, health, disability, sexuality, and particularly—as the majority of carers are women—gender (Greenwood et al. 2015; Sharma et al. 2016). Indeed, shouldering the burden of care can itself be understood as one of the ways that unjust social relations are made manifest: when health inequalities in racialized communities increase the likelihood for care to be required; when financial resources dictate competing responsibilities, access to private help, and the spaces where care takes place; or when women are expected to take disproportionate roles in caring for children or parents, over and above male partners or siblings (Ferrer et al. 2017; Funk et al. 2024, p. 7). Even for comparatively privileged carers, though, an additional layer of experience is imposed, which establishes its own (contingent and embedded) relationship with epistemic, health, and social justice. Interactions with services have their own vexed epistemological dynamics, and carers can be subject to specific forms of identity prejudice within them; this might manifest, as above, in medical attitudes to carers as anxious, difficult, or demanding non-experts, despite institutional commitments to knowledge sharing and ‘hearing carer voices’ (Brown^ 2021^; Lowndes et al. 2023, p. 100).

Here and elsewhere, there is a compelling case to consider epistemic injustice and effacement as an important and overlooked determinant of loneliness. In her famous account of hermeneutic injustice, Miranda Fricker explored the case of Carmita Wood, a victim of sexual harassment before (as Fricker has it) the epistemic resources existed to describe it. Wood, Fricker notes, was ‘deeply troubled, confused, and isolated’ by her inability to define precisely what had happened to her (Fricker, 2007, p. 151). Paul Crichton, Havi Carel, and Ian Kidd further suggest that ‘social isolation and epistemic impairment can be mutually reinforcing’, as ‘many of our epistemic practices are intrinsically social’ (Crichton, Carel and Kidd 2017, p. 69). More specifically, Brian Brown speculates that ‘blocks and barriers in knowledge [might] reflect something else, perhaps an absence of communitas or solidarity, or the loneliness of being a carer’ (Brown 2021, p. 65). Under this lens, we witness the lonely carer as alienated from their fellows but also, in the process, as alienated from the collective resources necessary to navigate opaque and isolating epistemic exchanges, or to understand their experiences as structural and shared (Hall 2017). This is usefully exemplified in a short conversation between one carer and the researcher interviewing, which bears repeating in full:

They did a survey, and in the survey, they were saying that people who were caring for children or adults with special needs were just being forgotten about. It showed you, on the Carers Week thing as well, teenagers and parents caring, and just saying, ‘We are just stuck in a corner. We cannot do anything.’

*Yes. How did that feel for you, to see all of that?*

It was actually good to see that there were other carers who felt the same way that we did. I thought it was just us, that we were being really hypersensitive about the whole thing.

*Aww. Is that really how you felt? Did you think it was just you?*

Yes, yes. I don’t have any other friends who have children with special needs, or children who are vulnerable. I was kind of the only one who felt it (P14W11).

For Olivia Sagan, epistemic injustice is a ‘potent mechanism via which to unpick how one can be silenced, invalidated and set adrift from trust, reciprocity, and rootedness in the world’ (Sagan 2020, p. 244). As Mary Larkin and Alisoun Milne have argued, these questions are central to the future of carer empowerment and thriving: ‘disempowerment occurs as a result of the knowledge of those subject to power being subordinated to the knowledge of those who have power. This is one of the most profound ways in which carers remain marginalised, have few choices and receive limited support from welfare agencies (Larkin and Milne 2014, p. 33).

### The historical bounds of carer loneliness

Fully understanding this nexus of loneliness, solitude, shame, and epistemic disempowerment requires an even longer lens than the immediate material and rhetorical conditions of care. In her classic genealogical analysis of the discourse of informal care in the UK, Janet Heaton shows how carers—as we now know them—were made visible (and to a degree co-created) by a nexus of services, carer organisations, pressure groups, and policy initiatives in the 1970s, with wider public recognition following in the 1980s and 1990s (Heaton 1999).

Heaton is not quite right to say that carers were ‘unheard of’ before the 1970s; more a problem of wording than her argument overall (Heaton 1999, p. 759). Indeed, it was in discussions of the social and financial toll that caring took that carers as a class had begun to be sketched out. For example, in a 1955 Sunday Observer article, ‘Forgotten Women’, Frances Thornton wrote that ‘old people are often referred to as a national problem: but how many of us also realise that they are an acute personal problem for hundreds of middle-aged women in this country, who are on duty day and night with their old folk, and whose health and finances are being ruined thereby’. Thornton depicted lives ‘filled with fear and worry’, where the ‘long, broken nights and the monotony and nervous strain of the days’ dovetailed into significantly constrained—and sometimes entirely stifled—opportunities for socialising, ‘much-loved work’, or religious observance (Thornton 1955).

Early journalistic exposés of carer loneliness took place in the context of a wider social and cultural crisis of connection, a formulation which foreshadowed (and necessarily complicates) analogous languages of epidemic loneliness in the present day (Cooper 2023). As part of a broader project of uncovering hidden experiences of loneliness in British society in the 1950s, 1960s, and 1970s, carers were identified—alongside groups such as older adults, students, and housewives—as being at particular risk. A 1973 article in the Daily Mirror, ‘Daughters of Loneliness’, made the connection particularly stark: ‘there are some 300,000 such daughters in this country—women who have sacrificed marriage and a career to stay at home and look after aged and ailing parents. Women who, too often, find themselves in poverty and, towards the end of their days, alone and unwanted’. The author, Donald Gomery, quoted Roxanne Arnold, the director of the National Council for the Single Woman and her Dependents: ‘How often do you hear of a son being asked to give up marriage or his job to do the same thing?’ Summing up, he noted that ‘the irony is that these daughters save the country millions of pounds… yet their reward is often poverty—and loneliness (Gomery 1973).

As was the case in 2020, the language of forgetting had a clear resonance here. But on what terms, precisely, were carers forgotten? With male carers barely—if at all—in cultural view (Arber and Gilbert 1989), these discussions took place in the context of a complex public reckoning over gender, fulfilment, and health, which played out primarily across discussions of work, education, domesticity, motherhood, marriage, loneliness, neurosis, suicide, and the built environment (Hayward 2007; Langhamer 2016; Tinkler et al. 2016; McCarthy 2020). From a plurality of competing aetiologies and anxieties, including oppositional discourses which placed maternal loneliness in explicit tension with the assumed loneliness of children whose mothers went out to work, feminists, doctors, and social scientists negotiated a hesitant consensus rooted in the language of balance between work and ‘home’, the latter envisaged for women as a mixture of housework, motherhood, and marriage (Cooper 2016). Although subject to extensive criticism by subsequent generations of feminists, who noted the contradictions and strain that it placed working mothers under, this nonetheless represented a fundamental conceptual redrawing of what women needed to be well. In the process of staking out civic and professional participation as key determinants of health, however, women with heightened caring responsibilities were deliberately and repeatedly excluded, in a long and slow death by caveat. For the sociologist Ferdinand Zweig, for example, work offered a number of considerable emotional and social affordances, but every woman’s ‘fund of energy’ had to be ‘judged against the background of the obstacles she has to overcome’ (Zweig 1952, p. 16). These could include the condition of the home she was expected to manage, the health and helpfulness of her husband, the number of her children, and whether they required special care:

If the child is sick, very delicate or an invalid, or needs special care or diet, obviously, no one can adequately replace the mother’s care, and her place is at home… the rule that proper arrangement can satisfactorily replace a mother’s care applies only to normal and healthy children both physically and emotionally (Zweig, 1952, p. 75).

Speaking at a conference on nursery provision in 1960, the child psychiatrist William Lumsden Walker concurred. The problem of generalised statements about working motherhood, he argued, was that ‘you produce rules which do not fit everybody’ (Lumsden Walker 1960, p. 12). Psychologists who opposed women’s work on child developmental grounds overlooked the ability of stable and happy families to endure any challenge: ‘these people can probably do almost anything they like, including going out to work, and nobody is going to suffer at all’ (Lumsden Walker 1960, p. 17). On the other hand, when ‘instability or serious insecurity’ entered the picture, it was necessary to be ‘very careful indeed’; in particular, mothers with children who were ‘sick or handicapped’ could not be ‘spared from the home’ (Lumsden Walker 1960, p. 13). Following the applause at the end of the address, the chair noted that he had ‘seen heads all the time nodding here and there’ (Fletcher 1960, p. 17). My point here isn’t that an otherwise progressive intellectual movement in the midst of significant political and cultural advances necessarily imposed an alien or unwelcome logic on carers. Quite the opposite, the conditions they laid down were already a lived reality for women whose economic situations allowed it. As Roxanne Arnold alluded, the gendered politics of caring underscored just how fragile feminist gains were (and are today). Buttressed by inter-war research on the psychological effects of mass unemployment, men’s claims to a right to work were ideologically unassailable, but women’s claims were fragile and comparably new, accepted with inconsistency, caution, and sufferance (Gomery 1973; Silverman et al. 2020). In the process of explaining why she felt ‘on the margins’ in 2020, one of our carers trailed off mid-sentence. ‘I’m a nurse’, she said. ‘I had to give up my nursing career, but nobody thinks about that’ (P8W10).

What post-war negotiations over the exceptionalism of care did was situate carers explicitly outside of shared political, cultural, and medical visualisations of human happiness and health. ‘Forgotten Women’ and ‘Daughters of Loneliness’ were each part of a wider (and persisting) tradition of writing about carer loneliness in managerial and fatalist terms, drawing attention to the problem but doing little to question its root causes. Even as journalists reported on significant suffering and harm, they helped naturalise the experiences they described; simultaneously, their contemporaries worked on new imaginaries of the healthy, connected, and fulfilled life, which contrasted distinctly with what carers could expect, an imaginative lacuna which desensitises publics and policymakers to their problems and severely constrains the scope of collective ambition in addressing them.

## Conclusion

The interviews our research team conducted with carers in 2020 distilled these unresolved tensions, one of many examples of the COVID-19 pandemic landing in ways that reproduced and elaborated far longer histories of exclusion and injustice. Such histories continue to interpolate themselves violently into the present, following carers and disabled people forward to the withdrawal of COVID adjustments when the pandemic was deemed, again following profoundly ableist calculations, to be over (McDermott 2023, p. 11). In narrating complicated experiences and lives, our carers pulled focus to the messy complexity of a series of overlapping failures and harms. They described feelings of loneliness, shame, and abandonment, which were framed and inflamed by the immediate circumstances of quarantine but rarely originated with them; which puncture sanitised and counter-political depictions of loneliness as something which ‘doesn’t discriminate’, alighting instead on its systemic and epistemic dimensions (Wilkinson 2022). Their accounts chime with research on loneliness and care (Vasileiou et al. 2017) and caring epistemologies (Brown 2021), which have begun to trace the entanglements between carer knowledge and carer isolation, but allow for a combined analysis which moves understandings of this relationship significantly forward.

In the context of a long economic, social and medical reliance on their unpaid labour, and where the only immediate practical alternatives frequently feel uncaring or care-less, this article has explored how carers are figured as a particular kind of shamed and devalued worker, with loneliness as a regrettable occupational disease (Lynch 2007; Isham et al. 2020, p. 85; P5W2). At the same time, carers have been set explicitly outside of rights and protections, such as they are, for more formalised work (Streeter 2023, p. 81). While carers advocate for themselves in important ways, these rarely trouble the crucial contract—that, in providing care, considerations of personal welfare become secondary (Vasileiou et al. 2017, p. 5). Shame, in particular, plays a vital disciplinary role here, restricting what it is socially and politically possible to do and say; as Brown has it, causing ‘striations in [the] epistemic topography, such that some things are more legitimate in the public domain than others’ (Brown 2021, p. 63). Carers speak about their own abandonment, but being seen to—or even seen to want to—relinquish care is frequently interpreted as a normative and ethical rupture, even in the face of significantly deteriorating physical and mental health on the part of the carer (Verbakel 2014). Thinking critically and expansively about carer loneliness, and the complex systems of abandonment, shame, and epistemic harm it takes place in, can take us right to the heart of the problem. Resisting a depoliticised – and so, of course, profoundly political—imagining of what loneliness is and where it comes from, using historical and humanities methodologies to excavate, interpret, and critique the terms and context of the phenomena we explore, and practising epistemic care in our own research, can help inform approaches to carer loneliness which are at least clear-sighted over the depth and scale of obstacles to overcome.

## Data Availability

The dataset analysed during this study is not available due to ethical concerns.

## Footnotes

1 I interpret this as a reference to the use of animals in studies on maternal/infant attachment and absence—see, for example, Robledo et al. 2022.

2 Almost certainly a reference to the film Cast Away (2000), a retelling of Robinson Crusoe in which Tom Hanks’ marooned FedEx employee makes an ersatz friend, Wilson, from a baseball.

3 This was a likely reference to Mina Akhtar’s *Guardian* article on the 10th of June 2020: ‘Stop calling family carers a ‘hidden army’. We’re not invisible, just ignored’ (Akhtar 2020).
